# Targeted metabolomic profiling in rat tissues reveals sex differences

**DOI:** 10.1038/s41598-018-22869-7

**Published:** 2018-03-16

**Authors:** Margherita Ruoppolo, Marianna Caterino, Lucia Albano, Rita Pecce, Maria Grazia Di Girolamo, Daniela Crisci, Michele Costanzo, Luigi Milella, Flavia Franconi, Ilaria Campesi

**Affiliations:** 1 Dipartimento di Medicina Molecolare e Biotecnologie Mediche, Università degli Studi di Napoli, “Federico II”, Napoli, Italy; 2 CEINGE Biotecnologie Avanzate, Napoli, Italy; 3 Associazione Culturale DiSciMuS, RFC 80026 Casoria, Napoli Italy; 4 Dipartimento di Scienze, Università degli Studi della Basilicata, Potenza, Italy; 5 Dipartimento di Scienze Biomediche, Università degli Studi di Sassari, Sassari, Italy; 6 Assessorato alle Politiche della Persone della Regione Basilicata, Potenza, Italy

## Abstract

Sex differences affect several diseases and are organ-and parameter-specific. In humans and animals, sex differences also influence the metabolism and homeostasis of amino acids and fatty acids, which are linked to the onset of diseases. Thus, the use of targeted metabolite profiles in tissues represents a powerful approach to examine the intermediary metabolism and evidence for any sex differences. To clarify the sex-specific activities of liver, heart and kidney tissues, we used targeted metabolomics, linear discriminant analysis (LDA), principal component analysis (PCA), cluster analysis and linear correlation models to evaluate sex and organ-specific differences in amino acids, free carnitine and acylcarnitine levels in male and female Sprague-Dawley rats. Several intra-sex differences affect tissues, indicating that metabolite profiles in rat hearts, livers and kidneys are organ-dependent. Amino acids and carnitine levels in rat hearts, livers and kidneys are affected by sex: male and female hearts show the greatest sexual dimorphism, both qualitatively and quantitatively. Finally, multivariate analysis confirmed the influence of sex on the metabolomics profiling. Our data demonstrate that the metabolomics approach together with a multivariate approach can capture the dynamics of physiological and pathological states, which are essential for explaining the basis of the sex differences observed in physiological and pathological conditions.

## Introduction

Numerous sex differences in the epidemiology, prevention, natural history, therapy and outcomes of diseases have recently been recognized^1,2^. Cardiovascular diseases, diabetes mellitus, renal and liver diseases are strongly influenced by sex^1–3^. Furthermore, sex differences in metabolic pathways, including lipid metabolism, have been reported^4,5^. Many sex differences appear to be organ- and parameter-specific^6–8^ and, therefore, they have to be identified as single parameters in individual organs. Rats with mice are the most used animals in preclinical pharmacological and toxicological tests^9^. Therefore, it is relevant to know if sex differences exist in rats and if such differences can be translate into humans.

The exact influence of sex on carnitine and its acyl esters (acylcarnitines, ACs) is not known, although some sex differences have been described, as in the case of free carnitine (C0) in plasma, liver, heart and skeletal muscle of pre-weaning rats^10^. In addition, C0, hexanoylcarnitine (C6), dodecanoylcarnitine (C12), tetradecanoylcarnitine (C14), hexadecanoylcarnitine (C16), octadecanoylcarnitine (C18), octadecenoylcarnitine (C18:1), and octadecadienoylcarnitine (C18:2) are lower in the male than in the female heart^11^, while octanoylcarnitine (C8), hexadecenoylcarnitine (C16:1), octadecatrienoylcarnitine (C18:3), and some long chain acylcarnitines (>C20) have similar levels in both sexes in Wistar rats^11^.

Carnitine and ACs are essential for the metabolism of fatty acids because they are vital for the transport of fatty acids across the mitochondrial membrane for beta oxidation, which produces acetyl-Coenzyme A and chain-shortened acyl products to preserve cellular coenzyme A (CoA) homeostasis^12,13^. ACs are very relevant in tissues characterized by high rates of beta oxidation, such as cardiac muscle, in which approximately 70–80% of the energy requirements are derived from fatty acid oxidation^14^.

Notably, C0 and ACs are dependent upon amino acids. In particular, endogenous carnitine synthesis, which mainly occurs in the liver and kidneys, requires the methylation of lysine (Lys) and methionine (Met) residues^15^. Propionylcarnitine (C3), isovalerylcarnitine (C5), and methylmalonyl carnitine (C4DC) are derived from the branched-chain amino acids isoleucine (Ile) and leucine (Leu)^16^, and together with the aromatic amino acids tyrosine (Tyr) and phenylalanine (Phe), these compounds are significantly associated with the prevalence and incidence of diabetes mellitus^16^. However, other intermediates can produce ACs^7,16^, whereas other tissues, such as skeletal muscle and heart acquire C0 from the blood^15,17^.

Considering that sex differences influence pathophysiology, disease outcomes, therapy, drug discovery and development, including preclinical tests, which very often utilize rats^18,19^, it is important to know whether sex affects the profile of carnitine and ACs in the liver, heart and kidneys of rats in order to have a powerful approach in examining intermediary metabolism. To gain a deeper insight into the sex-specific activities of the liver, heart and kidney, we use a targeted MS-based metabolomic platform to measure 13 amino acids and 37 ACs in healthy male and female rats while lysine (Lys), a precursor of carnitine^16^, was measured by HPLC. Target metabolomic profiling is one of the scientific approach to characterize biological systems, which ensure versatility, speed and reproducibility especially in quantitative analysis, allowing the analysis of a large number of samples. The data were collected at the same time and from the same animals and was integrated with several multivariate methods (cluster analysis, principal component analysis (PCA), linear discriminant analysis (LDA)) and linear correlation models to assess the correlations among the analysed molecules to ameliorate our knowledge on sex differences.

## Results

### Weights of animals and protein contents

The male and female rats enrolled were age-matched (7 weeks). As expected, females showed a significantly lower body weight than males (227 ± 13 g for males and 195 ± 10 g for females; P < 0.001). We chose age-matched animals with different body weights because age exerted important effect on metabolomics^20,21^. The total protein content was measured in each samples (15 for each sex and for each organ), and no statistical significant sex differences were detected. The protein abundance was 6.30 ± 2.27 and 6.53 ± 2.48 mg/ml for male and female livers, respectively; 11.90 ± 3.90 and 14.10 ± 2.90 mg/ml for male and female hearts, respectively; and 12.59 ± 0.91 and 17.52 ± 9.33 mg/ml for male and female kidneys, respectively. Metabolomic data were normalized to the total tissue protein content.

### Intra-sex and inter-organ analysis of amino acids

**Figure 1:**
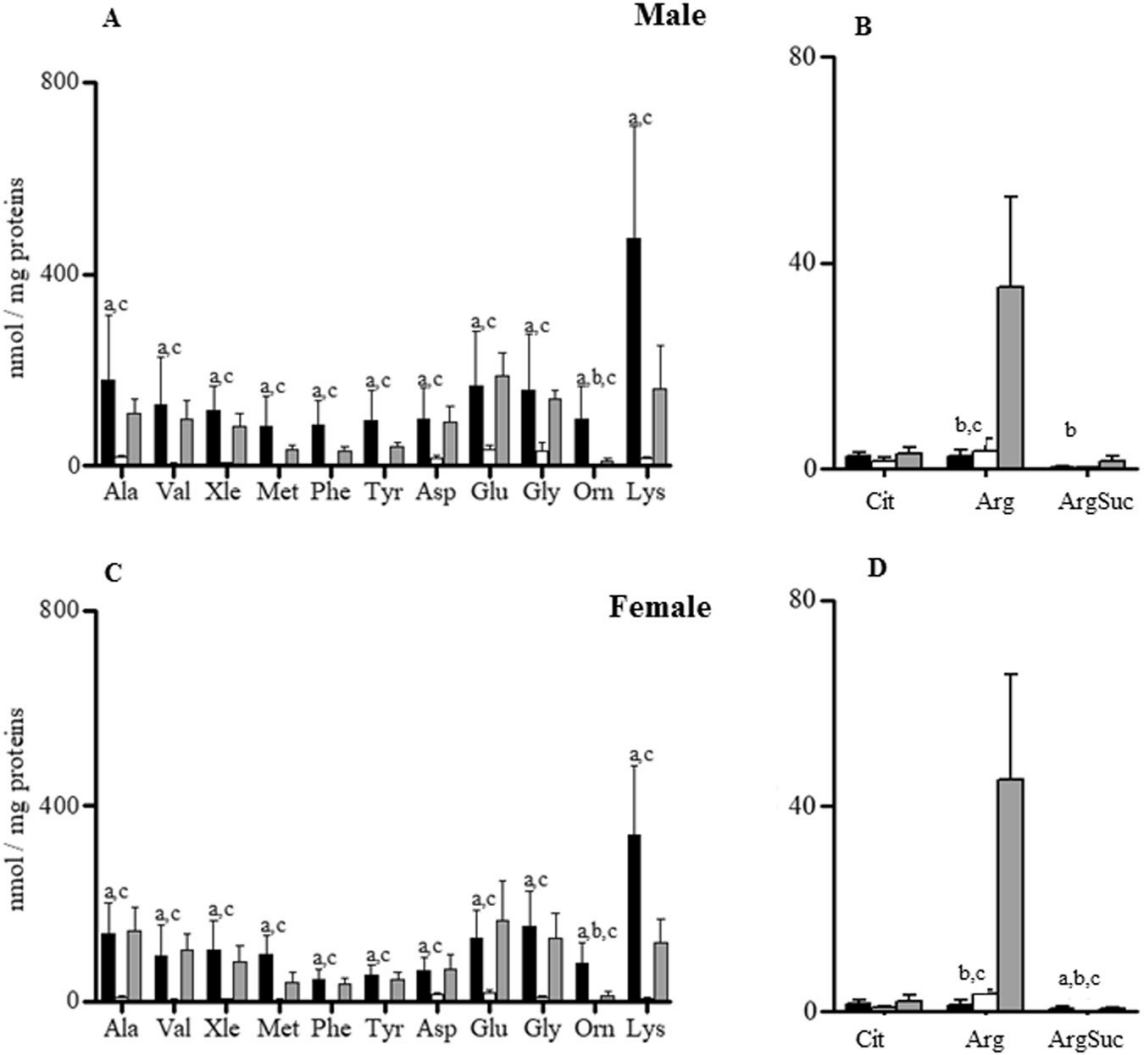
Intra-sex analysis of amino acids in livers (black bars), hearts (white bars) and kidneys (grey bars) of male (M) and female (F) rats. Data are the medians ± MAD of 15 samples for each tissue and sex. ^a^P < 0.05 between liver and heart; ^b^P < 0.05 between kidney and liver; ^c^P < 0.05 between heart and kidney.

In male cardiac ventricles, the concentrations of alanine (Ala), valine (Val), Leu + Ile (Xle), Met, Phe, Tyr, aspartate (Asp), glutamate (Glu), glycine (Gly), ornithine (Orn), and Lys were significantly lower than in the two other tissues (Fig. 1A). Conversely, the renal levels of arginine (Arg) and argininosuccinate (ArgSuc) were significantly higher than those in the liver and heart (Fig. 1B). The renal Orn concentration was significantly lower than that of the liver. Finally, the citrulline (Cit) abundance did not differ among the analysed tissues (Fig. 1B).

In female rats, overall, the cardiac amino acids levels were significantly lower than in the two other female tissues (Fig. 1C). Cardiac and hepatic Cit and Arg did not differ in a statistically significant manner, but Cit showed a significant intra-sex difference between the heart and kidney. Arg was significantly higher in kidneys than in livers and hearts, and Orn was significantly higher in livers than in kidneys (Fig. 1D).

### Inter-sex analysis of amino acids

**Figure 2:**
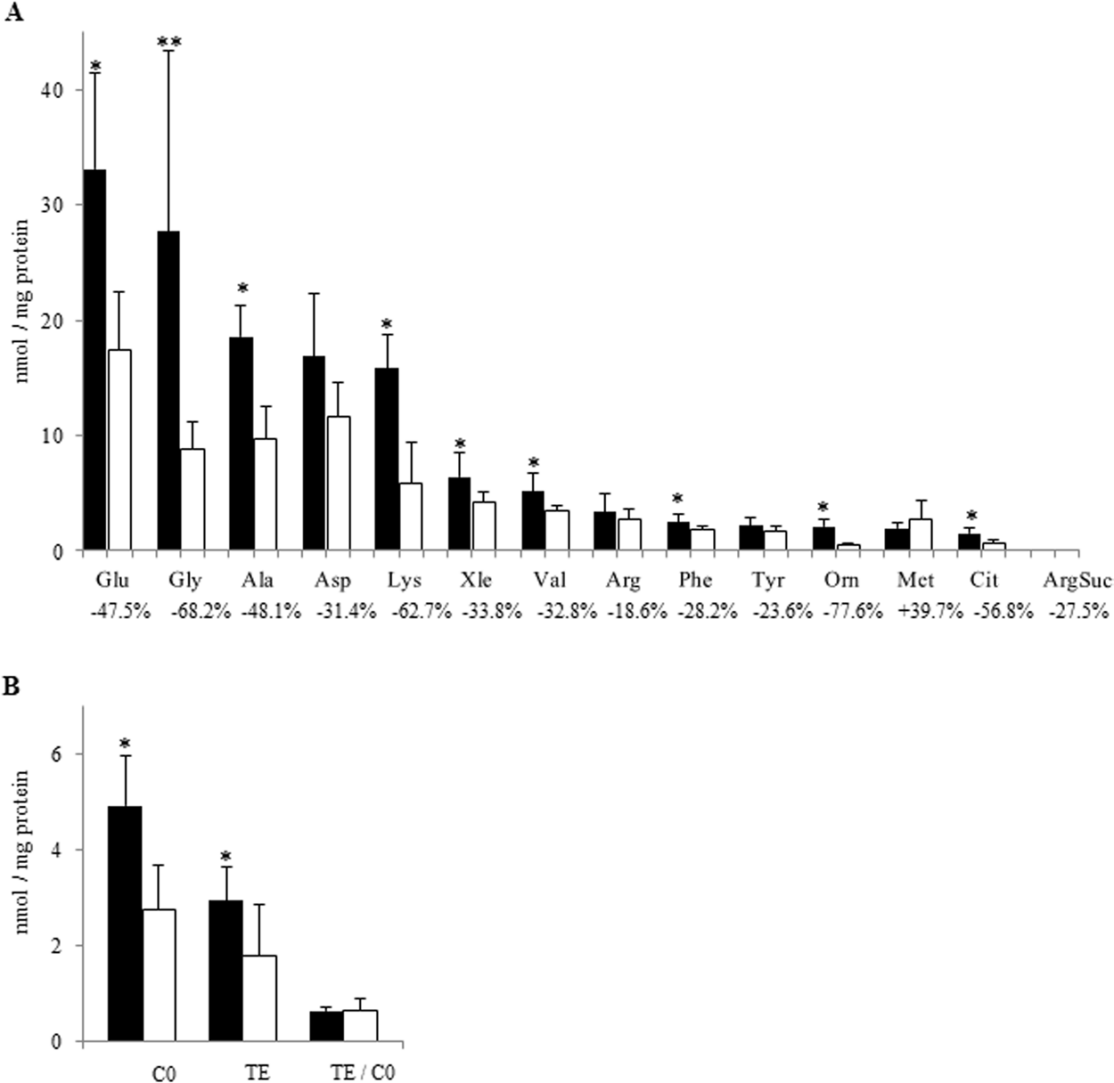
(**A**) Amino acids levels in male (black bars) and female (white bars) hearts. (**B**) C0, TE and TE/C0 ratio in male (black bars) and female (white bars) hearts. Data are the medians ± MAD of 15 independent samples for each sex. *P < 0.05 and **P < 0.001, versus females. Percentage of variation in females is reported under amino acid names.

Livers did not display significant sex differences in amino acids content (data not shown). In renal tissue, only ArgSuc showed a significant difference between males and females (1.95 ± 1.01 and 0.68 ± 0.36 nmol/mg proteins for males and females, respectively; P = 0.025, N = 15 for each experimental group). Moreover, in cardiac ventricles Glu, Gly, Ala, Xle, Val, Phe, Orn, Cit and Lys were significantly higher in male hearts than in female ones (Fig. 2A). By contrast, Asp, Arg, Tyr, Met, and ArgSuc did not display any significant sex differences (Fig. 2A).

### Intra-sex and inter-organ analysis of ACs

**Figure 3:**
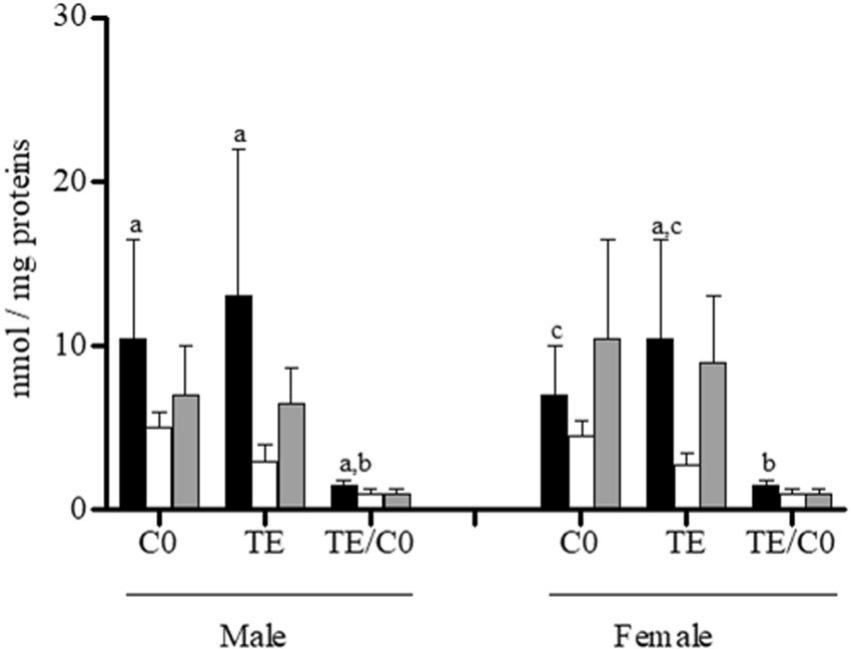
Intra-sex and inter-organ analysis of C0, TE and TE/C0 ratio in male and female livers (black bars), hearts (white bars) and kidneys (grey bars). Data are the medians ± MAD of 15 samples for each tissue and sex. ^a^P < 0.05 between liver and heart; ^b^P < 0.05 between kidney and liver; ^c^P < 0.05 between heart and kidney. ^a^P < 0.05 between liver and heart; ^b^P < 0.05 between liver and kidney; ^c^P < 0.05 between heart and kidney.

**Figure 4:**
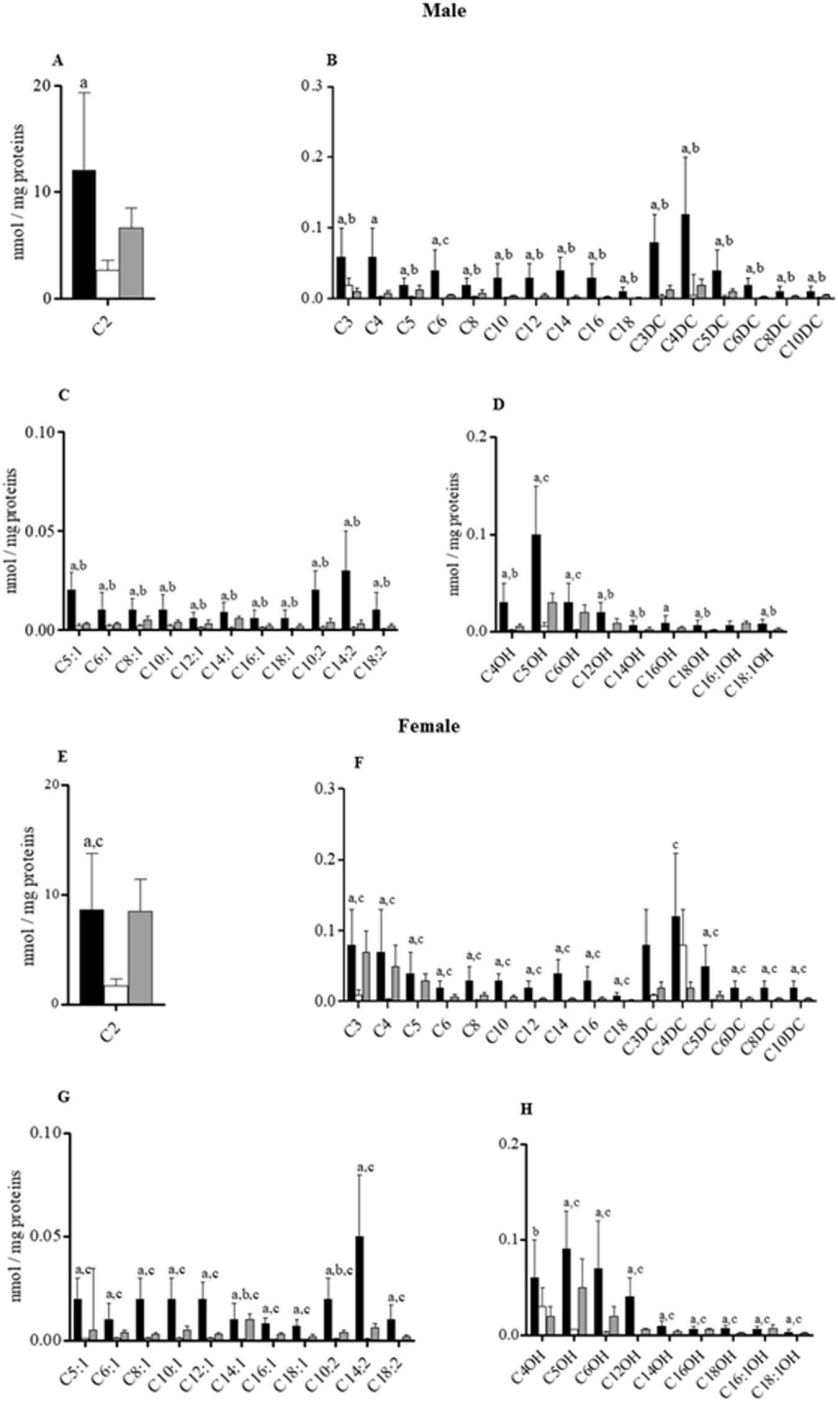
Intra-sex and inter-organ analysis of ACs in livers (black bars), hearts (white bars) and kidneys (grey bars) of male (M) and female (F) rats. Data are the medians ± MAD of 15 samples for each tissue and sex. ^a^P < 0.05 between liver and heart; ^b^P < 0.05 between liver and kidney; ^c^P < 0.05 between heart and kidney.

In males, C0 and total esterified carnitines (TE) were significantly lower in the heart than in the liver, whereas the TE/C0 ratio was significantly higher in livers than in the two other tissues (Fig. 3). In male rats, the intra-sex analysis of ACs revealed that among saturated carnitines, acetylcarnitine (C2) and C5 were significantly lower in the heart, whereas the concentrations of C3, butyrylcarnitine/isobutyrylcarnitine (C4), the fatty acid derived C6 and octanoylcarnitine (C8), decanoylcarnitine (C10), C12, C14, C16, C18, malonylcarnitine (C3DC), C4DC, glutarylcarnitine (C5DC), methylglutarylcarnitine (C6DC), octanedioylcarnitine (C8DC), and decanedioylcarnitine (C10DC) were significantly higher in the livers than in the two other tissues (Fig. 4A,B). The concentrations of the unsaturated carnitines tiglylcarnitine (C5:1), hexenoylcarnitine (C6:1), octenoylcarnitine (C8:1), decenoylcarnitine (C10:1), dodecenoylcarnitine (C12:1), tetradecenoylcarnitine (C14:1), C16:1, C18:1, decadienoylcarnitine (C10:2), tetradecadienoylcarnitine (C14:2), and C18:2 were significantly higher in the livers than in the 2 other tissues (Fig. 4C). The hydroxylated carnitines 3-hydroxybutyrylcarnitine (C4OH) which derived from ketone bodies^22^, 3-hydroxydodecanoylcarnitine (C12OH), 3-hydroxytetradecanoylcarnitine (C14OH), 3-hydroxyoctadecenoylcarnitine (C18:1OH), and 3-hydroxyoctadecenoylcarnitine (C18:1OH) (Fig. 4D), 3-hydroxyisovalerylcarnitine/3-hydroxy-2-methylbutyrylcarnitine (C5OH), 3-hydroxyhexanoylcarnitine (C6OH), and 3-hydroxyhexadecenoylcarnitine (C16:1OH) had significantly lower values in male hearts than in livers and kidneys (Fig. 4D); and 3-hydroxyhexadecanoylcarnitine (C16OH) was significantly lower in the heart in comparison with the liver (Fig. 4D).

In female tissues, C0 was significantly lower in cardiac ventricles than in kidneys; the TE content had the lowest value in hearts in comparison with livers and kidneys, whereas the TE/C0 ratio was significantly higher in livers than in the other two tissues (Fig. 3). In females, the levels of C2, C3, C4, C5:1, C5, C6, C5OH, C8, C10:1, C10, C5DC, C12:1, C12, C6DC, C14:2, C14:1, C14, C8DC, C16:1, C16, C10DC, C16OH, C18:1, C18, C18:1OH, C6OH, C6:1, C8:1, C10:2, C12OH, and C14OH were lower in female hearts than in livers and kidneys (Fig. 4E–H). C4DC had the lowest value in kidney compared to livers and hearts, whereas C12:1 was also significantly higher in livers than in kidneys, as was C4OH (Fig. 4F). These data demonstrated that males and females showed lower levels of carnitines in the heart.

### Inter-sex analysis of carnitines and ACs

The level of C0, TE or in the TE/C0 ratio did not diverge between males and females in the liver and kidney (data not shown). In contrast, in cardiac ventricles, C0 and TE were significantly higher in males than in females, but the TE/C0 ratio did not present a sexual dimorphism (Fig. 2B).

**Figure 5:**
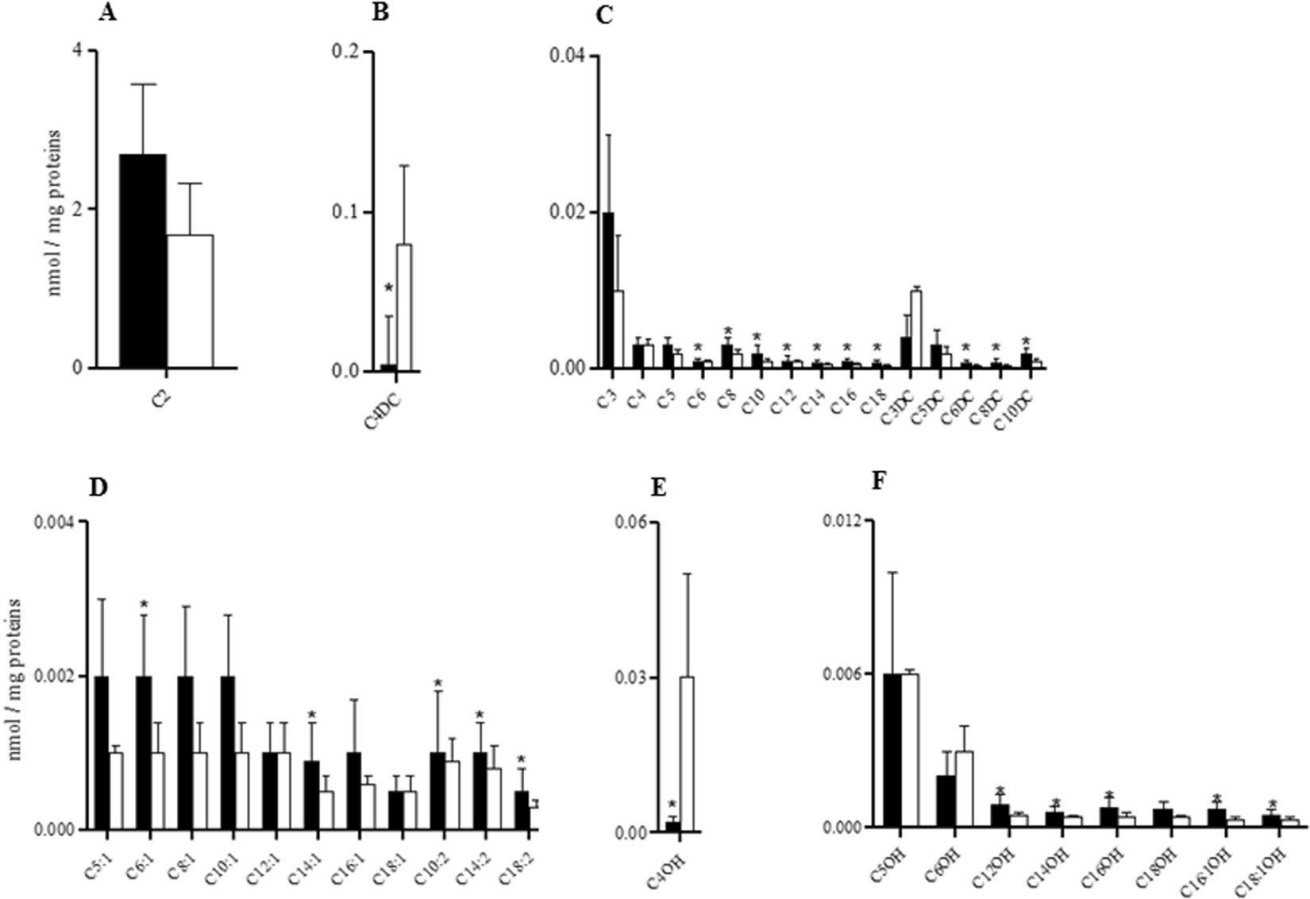
Inter-sex analysis of AC levels (nmol/mg protein) in male (M) and female (F) rat hearts. Values are the medians ± MAD of 15 independent samples for each sex. *P < 0.05 versus females.

At cardiac levels, C2, C6, C8, C10, C12, C6DC, C14:2, C14:1, C14, C8DC, C16, C10DC, C16OH, C18, C18:1OH, C6:1, C10:2, C12-OH, C14-OH, C16:1OH, and C18:2 were significantly higher in males than in females (Fig. 5A–F). The differences ranged from −10% to −57.1%. Only C3DC, C4DC, and C4OH were higher in female hearts than in male ones (Fig. 5C–E), but the sex differences were very large. In particular, C4DC, C4OH and C3DC were increased by approximately 1233%, 900%, and 150% respectively. C3, C4, C5, C5DC, C5:1, C8:1, C10:1, C12:1, C16:1, C18:1, C5OH, C6OH, and C18OH did not present significant sex differences (Fig. 5A–F).

Regarding renal levels, the majority of the saturated, unsaturated and hydroxylated carnitine (measured in 15 animals of a single sex and expressed as nmol/mg protein) did not generally differ between males and females, with the exception of C3 (0.013 ± 0.006 and 0.07 ± 0.03 for males and females, respectively; P < 0.001), C4 (0.008 ± 0.003 and 0.05 ± 0.03 for males and females, respectively; P < 0.001), C5 (0.01 ± 0.006 and 0.03 ± 0.01 for males and females, respectively; P = 0.022), C10 (0.004 ± 0.001 and 0.007 ± 0.03 for males and females, respectively; P = 0.047), C5:1 (0.003 ± 0.00 and 0.005 ± 0.002 for males and females, respectively; P = 0.009), and C4OH (0.006 ± 0.002 and 0.02 ± 0.01 for males and females, respectively; P < 0.001), which were significantly higher in female kidneys than in male kidneys.

### Correlation analysis between amino acids and carnitines

C0 is derived from Met and Lys^23^; therefore, we looked for the correlation between Met and Lys and C0. In all organs of both sexes, C0 and Met were significantly and positively correlated (Table 1). In contrast, Lys was not related to C0 levels in any organ of either sex (data not shown). Some carnitines such as C3, C5 and C4DC are derived from branched chain amino acids^24^; therefore, the association between them and Val and Xle was also evaluated. C3 was positively correlated with Val and Xle in male and in female livers and in the female hearts but not in kidneys (Table 1). In livers and hearts, Xle and Val were positively correlated with C5 in males and in females. In kidneys a significant positive correlation was detected only in females (Table 1). Finally, C4DC was positively correlated with Val and Xle in male and in female livers and kidneys and with Val and Xle only in female cardiac tissue (Table 1).

**Table 1:** Analysis of correlations among levels of carnitines and amino acids in male (M) and female (F) rat organs.

	Sex	Liver	Heart	Kidney
C0 – Met	M	r = 0.835; P < 0.001	r = 0.718; P = 0.002	r = 0.837; P < 0.001
	F	r = 0.637; P < 0.001	r = 0.754; P < 0.001	r = 0.664; P = 0.006
	M			
C3 – Val	F	r = 0.694; P < 0.001	r = 0.671; P = 0.006	r = −0.305; P = 0.279
	M	r = 0.667; P < 0.001	r = 0.139; P = 0.611	r = 0.375; P = 0.162
	F			
C3 – Xle	M	r = 0.713; P < 0.001	r = 0.525; P = 0.043	r = −0.182; P = 0.521
	F	r = 0.663; P < 0.001	r = 0.307; P = 0.257	r = 0.475; P = 0.07
	M			
C5 – Val	F	r = 0.817; P < 0.001	r = 0.804; P < 0.001	r = 0.108; P = 0.704
	M	r = 0.617; P = 0.001	r = 0.546; P = 0.03	r = 0.746; P < 0.001
	F			
C5 – Xle	M	r = 0.854; P < 0.001	r = 0.625; P = 0.01	r = 0.103; P = 0.71
	F	r = 0.640; P < 0.001	r = 0.664; P = 0.006	r = 0.796; P < 0.001
	M			
C4DC – Val	F	r = 0.834; P < 0.001	r = 0.389; P = 0.146	r = 0.556; P = 0.037
	M	r = 0.693; P = 0.001	r = 0.514; P = 0.05	r = 0.618; P = 0.013
	F			
C4DC – Xle	M	r = 0.838; P < 0.001	r = 0.432; P = 0.104	r = 0.657; P = 0.001
	F	r = 0.698; P < 0.001	r = 0.654; P = 0.008	r = 0.536; P = 0.038

The Spearman Product Moment Correlation coefficient (r) and the P value are reported for each tissue and sex.

### Cluster analysis

**Figure 6:**
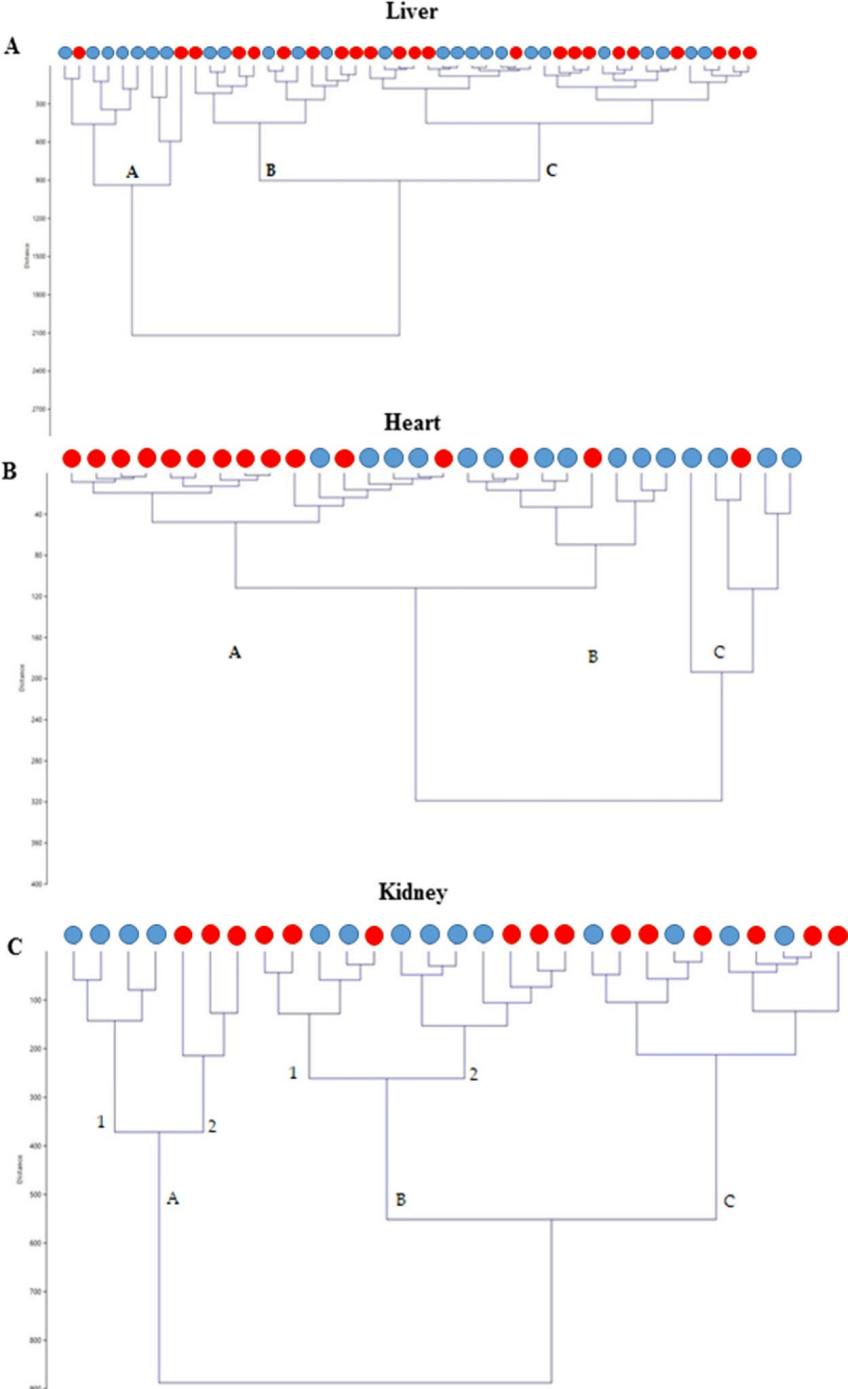
Cluster analysis depicting liver (**A**), heart (**B**) and kidney (**C**) sample distribution on the basis of Ward’s method used for hierarchical clustering. All dendrograms were constructed using together male and female samples per each organ metabolomic dataset. Male samples are depicted using a blue dot while female samples are in red.

In all organs, 3 main clusters were evidenced (Fig. 6). In the liver, the branch A contained all male samples and 2 female samples, while the remaining samples were not clearly separable indicating the widest distance (and the higher variability) in comparison with the heart and kidney (Fig. 6A). At cardiac level, the cluster analysis confirmed the results of univariate analysis showing a clear sex discrimination. In particular, branch C contained all male samples (with the exception of 1 female sample) and had the widest distance from branches A and B (Fig. 6B). The latter contained the most of the females and 4 male samples. In kidney, 2 sub-clusters in branch A (A1 and A2) divided samples per sex such as sub-branch 2B (Fig. 6C).

### LDA and PCA analysis

**Figure 7:**
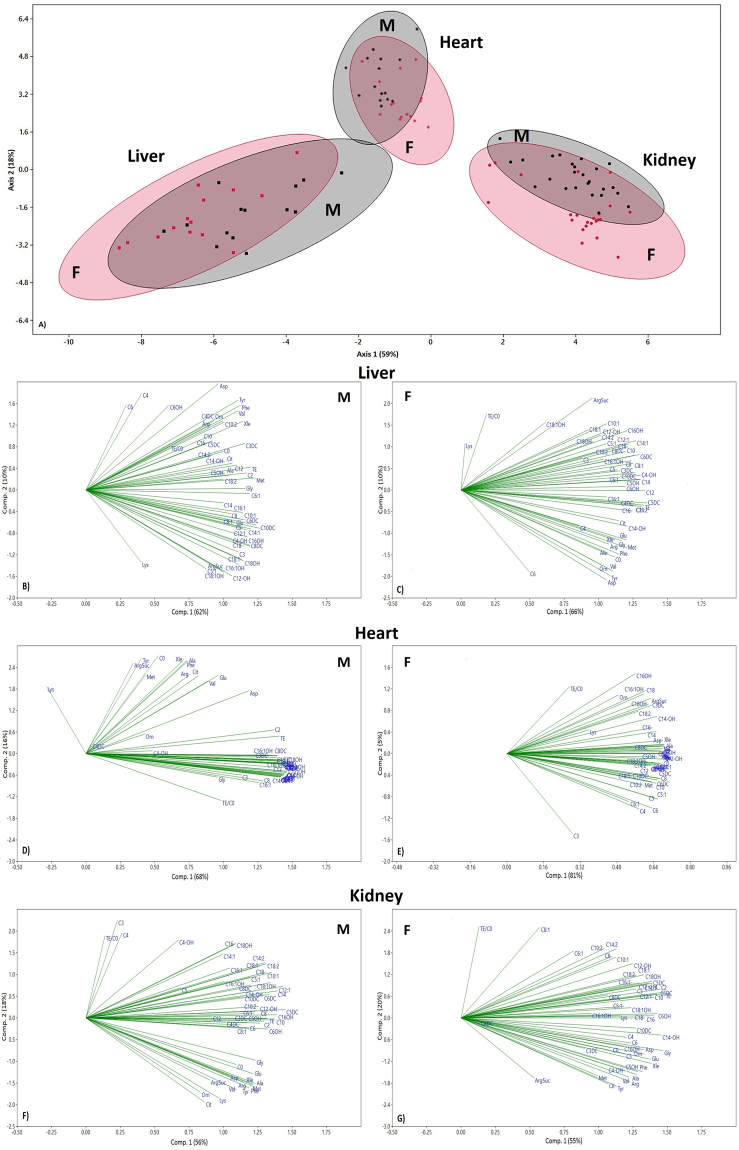
LDA score plot of all molecules (Panel A) and PCA of all molecules stratified according to sex and organ (panel B–F). Panel A, black and the red colours indicate male and female samples, respectively. Plotted ellipses estimate a region where 95% of population points are expected to fall. Panels B and C represent PCA loading plots in male and female liver samples; Panels D and E represent PCA loading plots in male and female heart samples; and Panels F and G represent PCA loading plots in male and female kidney samples.

LDA and PCA were performed including all measured parameters. In LDA analysis, organs and sexes were considered qualitative variables while tested compounds were considered quantitative variables. LDA analysis showed a clear organ clustering (Fig. 7A), while a significant separation between sexes was not evident. After stratification according to sex and organ, PCA revealed that the first (Comp 1) and second (Comp 2) principal components explained most of the variance in both sexes. In detail, in the male liver, Comp 1 and Comp 2 explained 62% and 10% of the variance, respectively and a total of 25 components were generated. Amino acids demonstrated a low loading on Comp 2, with the exception of Lys, which was negatively and strongly related to Comp 2 (Figs 7B, S1A). In the female liver, Comp 1 and 2 explained 66% and 10% of the overall variance, respectively, and a lower number (21) of total components versus males were generated (Figs 7C, S1B). Comp 1 included all tested variables on the positive side, with Lys, TE/C0, C6 and C18:1OH showing the highest positive score. Xle, Glu, Gly, Arg, Met, Ala, Phe, Orn, Val, Tyr, C0 and C6 were negatively correlated with Comp 2, whereas ArgSuc and Lys were positively related to Comp 2. In male and female hearts, the variance was explained by 14 principal components (Figs 5D,E, S1C,D). In males, Comp 1 and Comp 2 explained 68% and 16% of the overall variance, respectively, and almost all amino acids (except Orn and Gly) were responsible for the positive variance of Comp 2. In female hearts, all variables were positively related to Comp 1, and all showed a strong loading, with the exception of Lys, C3 and TE/C0. Finally, in male and female kidneys, the variance was explained by 13 principal components a value that was very similar to that observed in the heart (Figs 7F,G, S1E,F). In detail, in male kidneys, Comp 1 and Comp 2 explained 56% and 18% of the variance, respectively. TE/C0, C3, C4, C5 and C4OH showed a low loading on Comp 1, whereas all amino acids together with C0 were responsible for the negative variance of Comp 2. In females, numerous variables, except for C6:1, C8:1, ArgSuc and TE/C0, were positively related to Comp 1. Comp 2 mainly depended on amino acids that were all negatively correlated with it, with the exception of Lys.

## Discussion

Here, using a target metabolomic approach, we confirm that some amino acids and AC levels are organ-dependent in the rat heart, liver and kidney. We also show that they clustered and are related to each other in an organ- and parameter-specific manner. However, the major novelty is represented by the effect of sex on the amino acids and AC levels.

Globally, the male and female livers contain the highest amount of C0, ACs and amino acids, with some exceptions, while, male and female hearts contain the lowest levels of the studied parameters, with some exceptions. Lower cardiac levels of amino acids could be ascribed, at least in part, to the high energy demand necessary for the activity of heart^25^. The fact that the highest levels of Orn occur in the liver is not surprising because Orn plays a crucial role in ammonia detoxification, which occurs mainly in the liver^26^, whereas the highest renal level of Arg could be ascribed to the so-called “intestinal-renal axis”^27^.

The levels of C0 and TE are similar in livers and kidneys, the organs involved in the biosynthesis and elimination of carnitine^15^. The low level of cardiac ACs could depend on the fact that the energy requirements of the heart are mainly derived from fatty acid oxidation^14^. The high levels of cardiac mitochondrial carnitine acetyltransferase could influence the mitochondrial acetyl-CoA balance, converting short-chain acyl-CoA into its corresponding carnitine conjugates and permitting the efflux of excess acyl moieties. Indeed, the notion that mitochondria could behave differently in different organs is not new. In particular, cardiac acetyl-CoA seems to be less accessible to carnitine acetyltransferase, as acetyl-CoA is more tightly coupled to the tricarboxylic acid cycle than it is in the liver or kidney^14^. The organ differences observed in C0 and ACs suggest that mitochondrial function is organ-specific, as supported by proteomics studies^28^. Organ-specific differences in the metabolome have been already described in the brain, heart, kidney, and liver tissues of 26 mammalian species^29,30^.

Interestingly, females display higher renal levels of C3, C4, C5, C10, C5:1, and C4OH, whereas cardiac C3DC, C4DC and C4OH are much more elevated in females than in males. C3DC, derived from malonyl-CoA, is a key regulator of fatty acid oxidation and an inhibitor of carnitine palmitoyltransferase 1^31^, the rate-limiting enzyme for mitochondrial fatty acid uptake, and its inhibition leads to the reduction of fatty acid β-oxidation. C4DC is dependent on vitamin B12^32^, and the metabolism of methylmalonyl-CoA is inhibited in B12-deficient rats^33,34^. Finally, the dramatic increase in cardiac and renal C4OH suggests an impaired oxidation of hydroxybutyryl-CoA, an intermediate in ketone oxidation and a product of fatty acid oxidation. Furthermore, it has recently been suggested that C4OH could play a role in determining insulin resistance^16^, and interestingly, cardiac diabetic complications are more relevant in women than in men^3,35^. C3, C5, and C4DC are derived from branched chain amino acids Ile and Leu^16^, which, together with aromatic amino acids (Tyr and Phe), display a correlation with the onset of and predisposition to future diabetes mellitus type 2^16^, and this aspect could be more relevant in females than in males. In addition, recent data also highlight a positive association between the atrial concentrations of ketogenic amino acids and hydroxybutyrate and persistent atrial fibrillation^36^. In contrast, in mice, C0 and a small set of 17 ACs have been found to be more elevated in females than in males^37^. The discrepancy could depend on the animal species (mouse versus rat). Moreover, the animal strain could have some importance. For example, in the heart of Wistar rats, carnitine and C6, C12, C14, C16, C18, C18:1; and C18:2 are lower in the male than in the female heart^11^, while C8, C16:1, C18:3; C20, C20:1; C20:2, C20:4, C22, C22:1, C22:4 and C22:6 were similar in both sexes. Here, we show that the reverse is observed in Sprague Dawley rats for C6, C12, C14, C16, C18, C18:1 and C18:2. The discrepancy between Wistar and Sprague Dawley rats suggests the importance of strain for sex differences has already evidenced studying different parameters^38,39^.

Globally, each animal organ has a different metabolic profile that is influenced by sex in an organ- and parameter-specific way. The organ differences in carnitine and ACs probably reflect the different metabolic roles of each organ. Namely, cardiac mitochondria are specialized in ATP generation, primarily from fatty-acid oxidation, and the urea cycle is very relevant in hepatic mitochondria, whereas renal mitochondria seem to rely on the metabolism of some amino acids^40^. The influence of sex on organ-specific differences in metabolism confirms the importance of sex in understanding physiology and disease, as already shown in studies that examine different parameters.

As already mentioned, differences in the metabolome among different organs have been already described in the brain, heart, kidney, and liver tissues of 26 mammalian species^29,30^. However, these studies do not consider the influence of sex, as males and females are not separately analysed. Globally, the higher concentration of carnitines and amino acids in males may, at least partially, depend on an organ size that it is bigger in males than in females^37^. However, this reasoning does not seem to hold true because sex differences are not evident for all studied compounds and for all organs. The observed sex differences could partially depend on sex hormones^41,42^.

Interestingly, in all organs of both sexes, a positive correlation exists between Met (a precursor of endogenous synthesis of C0) and C0, whereas Lys (another precursor of C0 endogenous synthesis) is not associated with C0, suggesting a more relevant role of Met in the endogenous synthesis of C0. Interestingly, the strength of the correlations between Met and C0 depends on the sex and the organ. Val and Xle (both precursors of C5) are positively associated with C5 in all female organs, whereas in males, the correlation is present only in the liver and heart. Again, the strength of the correlations is organ-and sex-specific, as in the case of C3 and C4DC.

Cluster analysis identifies homogenous groups of observations only in the heart, clearly separating male from female animals, confirming univariate analysis. The variance among organs and sexes is mainly explained by PCA in that the first 2 components show a variance higher than 70% in each sex and in each organ. In particular, all variables contribute to Comp 1, whereas amino acids mainly contribute to Comp 2. LDA confirms the differences among organs being more effective in doing that than PCA, at least in our dataset but it is not effective in separating sexes.

The metabolic profiles seem to be specific to each tissue but males and females have a divergent sex-specific trajectory especially in the heart, suggesting that metabolomic analysis combined with multivariate analysis (cluster analysis, LDA and PCA) are effective tools for differentiating sex-and tissue-specific differences in rat organs, as previously shown in humans^43^. In particular, cluster analysis seems to be the most suitable method to evidence sex differences, at least in our samples.

This appears especially true at cardiac level and this are in line with the numerous differences observed in cardiovascular diseases^11,12^. Finally, these results provide a prospective resource for the study of pathophysiology and developing novel therapeutic targets in precision medicine. Knowledge of metabolomics can, in fact, help to identify biomarkers for disease and therapeutic efficacy and, as consequence, the determination and management of illness.

## Methods

### Animals

Male and female Sprague-Dawley rats (7 weeks old) were purchased from Harlan, Italy. Rats (2–3 per cage; males separated from females) were maintained on a 12 h light/dark cycle and were allowed food and water ad libitum until being sacrificed. All animals received the same food (standard commercial pelleted diet), the same water and the same housing conditions (included an adaptation period of 2 weeks before experiments). The experimental protocols were carried out in accordance with Italian law (DL 116, 1992) and the National Institutes of Health principles of laboratory animal care (NIH 80–33, revised 1996), and approved by the Independent Bioethics Committee for the Use of Experimental Animals at the University of Sassari (CIBASA). The sacrifice of the animals was performed in the morning for both sexes to avoid any variation due to time of sample collection. The animals were euthanized by decapitation, and the thoracic and abdominal cavities were opened to expose the hearts, livers and kidneys. The organs were rapidly removed and washed with ice-cold PBS. Parts of each tissue were weighed and homogenized in ice-cold PBS and stored at −80 °C.

### Metabolite measurements

Metabolites were extracted according to procedures already used for human tissues^44^. The frozen tissues were homogenized in 500 μL of 50:50 cold methanol/0.1 M HCl. The mechanical homogenization was performed using Dounce homogenizer, coupled with loose pestle. Proteins were separated by metabolite by centrifugation at 13,000 rpm, 60 min, 4 °C. The protein pellet was dissolved in 7 M urea, 2 M thiourea, 30 mM Tris-HCl, and 4% CHAPS, and the protein content was determined by Lowry assay^45^. The supernatant was collected and adjusted to pH 7–8 with KOH and re-centrifuged (40 min at 13,000 rpm). The supernatant was collected, dried under nitrogen and used for the subsequent analysis of amino acids and ACs. The dried supernatant was dissolved in methanol containing labelled standards. The standard concentrations were in the 500–2500 μmol/L range for amino acids and in the 7.6–152 μmol/L range for ACs. Labelled amino acids were ^15^N, 2-^13^C-Gly, ^2^H_4_-Ala, ^2^H_8_-Val, ^2^H_3_-Leu, ^2^H_3_-Met, ^13^C_6_-Phe, ^13^C_6_-Tyr, ^2^H_3_-Asp, ^2^H_3_-Glu, ^2^H_2_-Orn, ^2^H_2_-Cit, and ^2^H_4_,5-^13^C-Ar. The labelled carnitines were ^2^H_9_-C0, ^2^H_3_-C2, ^2^H_3_-C3, ^2^H_3_-C4, ^2^H_9_-C5, ^2^H_3_-C8, ^2^H_9_-C14, and ^2^H_3_-C16. After 20 min at room temperature on an orbital shaking system, the samples were dried under a nitrogen flow at 50 °C. The ACs and amino acids were derivatized to butyl esters with 80 μL of 3 N HCl in N-butanol at 65 °C for 25 min. After derivatization, the samples were dried under nitrogen flow at 50 °C and then re-suspended in 300 μL of acetonitrile/water (70:30) containing 0.1% formic acid. Forty μL were injected in the flow injection analysis mode for the MS/MS experiments. The analyte panel consisted of 14 amino acids and 37 ACs.

The reported amino acids, with exception of Lys, and ACs were measured by LC-MS/MS as previously described^46,47^. The analysis was performed using an API 4000 triple quadrupole mass spectrometer (Applied Biosystems-Sciex, Toronto, Canada) coupled with an 1100 series Agilent high-performance liquid chromatograph (Agilent Technologies, Waldbronn, Germany). Data were quantitatively analysed with ChemoView v1.2 software by comparing the analyte and its corresponding internal standard. The MS/MS analysis of Ala, Val, Xle, Met, Phe, Tyr, Asp, and Glu was performed by using a Neutral Loss of 102 Da scan function; the other amino acids (Orn, Cyt, Arg, ArgSucc) were analysed using a Multiple Reaction Monitoring (MRM) experiment. Finally, the AC analysis was performed by a Precursor Scan of 85 Da fragments.

The use of internal standardization employing stable isotope-labelled versions of the analytes allowed us to correct for the matrix effect. The accuracy and precision of the method are evaluated in each set of analyses using quality control (QC) samples prepared at four different concentrations (low, mid, high and very high). The quality control samples were provided by the Center for Disease Control and Prevention (Atlanta, GA, USA). Table S1 shows the QC analytical information. Lys, which is directly linked to carnitine metabolism was measured by HPLC as previously described^47,48^ with an Agilent Technologies 1200 Series LC System with an Agilent Zorbax Eclipse XDB-C18 analytical column (5 μm, 4.6 × 150 mm) and Agilent Eclipse XDB-C18 analytical guard column (5 μm, 4.6 × 12.5 mm). The amino acid was identified and quantified by its retention time and absorption ratio by comparison with the ratio of authentic compounds in the calibration solution. Metabolomic data were normalized by the tissue protein content. Quality controls were provided by ERNDIM (European Research Network for evaluation and improvement of screening, Diagnosis and treatment of Inherited disorders of Metabolism, Manchester, UK) (www.erndimqa.nl).

### Statistical analysis

For the statistical analysis of the data, males and females were compared. Data are reported as the median ± median absolute deviation (MAD). The distribution of samples was evaluated using the Kolmogorov–Smirnov and Shapiro tests. Continuous parametric variables were analysed using Student’s t-test. Non-parametric variables were compared with the Mann-Whitney rank test. An intra-sex analysis of the data was performed by one-way ANOVA on the ranks. The strength of the association between pairs of variables was evaluated by the Spearman Product Moment Correlation coefficient using the SigmaStat software. For all tests, a P value ≤ 0.05 was considered statistically significant.

Cluster analysis, a method for identifying homogenous groups of objects called clusters, was performed using Ward’s method. Observations in a specific cluster share many characteristics, but they are very different to objects not belonging to that cluster. Ward’s method for hierarchical clustering, which uses the F value to maximize the significance of differences between cluster, obtaining the highest statistical power, was undertaken in order to determine the number of clusters, or subgroups, present in the data^49^.

LDA looks for linear combinations of independent variables by means of a qualitative dependent variable and several quantitative independent variables, and it is also referred to as chemometric in the literature^50,51^. The generated confusion matrix (Table S1), which allows visualization of the performance of an algorithm, contains the reclassification of the observations (actual versus predicted) and allows to quickly see the percentage of well-classified observations (the ratio of the number of observations that have been well classified over the total number of observations). In our dataset, the confusion matrix was equal to 97% confirming the suitability of this method for metabolomic data analysis (Table S2).

PCA is an unsupervised multivariate statistical tool that analyses data sets consisting of a large number of variables to develop a new and easier model with a smaller number of artificial analyses that accounts for most of the variance in the data set. Data were normalized and used for PCA analysis. Samples were compared for male and female organs, and in the case that one sample value was not available, it was treated with a mean normalized value imputation.

Cluster analysis, PCA and LDA were computed using the statistical package Statistica for Windows (ver. 5.1., 1997) (Statsoft Inc., Tulsa, OK, USA).

## Electronic supplementary material

Supplementary tables and figure

## References

[1] Legato MJ (2017). Principles of gender-specific medicine. Gender in the genomic era. Boston.

[2] Regitz-Zagrosek V (2015). Gender in cardiovascular diseases: impact on clinical manifestations, management, and outcomes. Eur Heart J.

[3] Campesi I, Franconi F, Seghieri G, Meloni M (2017). Sex-gender-related therapeutic approaches for cardiovascular complications associated with diabetes. Pharmacol Res.

[4] Wang X, Magkos F, Mittendorfer B (2011). Sex differences in lipid and lipoprotein metabolism: it’s not just about sex hormones. J Clin Endocrinol Metab.

[5] Blaak E (2001). Gender differences in fat metabolism. Curr Opin Clin Nutr Metab Care.

[6] Addis R (2014). Human umbilical endothelial cells (HUVECs) have a sex: characterisation of the phenotype of male and female cells. Biol Sex Differ.

[7] Campesi I (2013). Glutamyl cycle in the rat liver appears to be sex-gender specific. Exp Toxicol Pathol.

[8] Campesi I, Straface E, Occhioni S, Montella A, Franconi F (2013). Protein oxidation seems to be linked to constitutive autophagy: a sex study. Life Sci.

[9] The society of toxicology. Animals in research. The importance of animals in the science of toxicology https://www.toxicology.org/pubs/docs/air/AIR_Final.pdf (1999).

[10] Borum PR (1978). Variation in tissue carnitine concentrations with age and sex in the rat. Biochem J.

[11] Devanathan S (2016). Sexual dimorphism in myocardial acylcarnitine and triglyceride metabolism. Biol Sex Differ.

[12] Sewell AC, Bohles HJ (1995). Acylcarnitines in intermediary metabolism. Eur J Pediatr.

[13] Steiber A, Kerner J, Hoppel CL (2004). Carnitine: a nutritional, biosynthetic, and functional perspective. Mol Aspects Med.

[14] Taegtmeyer H (1994). Energy metabolism of the heart: from basic concepts to clinical applications. Curr Probl Cardiol.

[15] Vaz FM, Wanders RJ (2002). Carnitine biosynthesis in mammals. Biochem J.

[16] Schooneman MG, Vaz FM, Houten SM, Soeters MR (2013). Acylcarnitines: reflecting or inflicting insulin resistance?. Diabetes.

[17] Vaz FM, Ofman R, Westinga K, Back JW, Wanders RJ (2001). Molecular and biochemical characterization of rat epsilon -N-trimethyllysine hydroxylase, the first enzyme of carnitine biosynthesis. J Biol Chem.

[18] Franconi F, Rosano G, Campesi I (2014). Need for gender-specific pre-analytical testing: the dark side of the moon in laboratory testing. Int J Cardiol.

[19] Zakiniaeiz Y, Cosgrove KP, Potenza MN, Mazure CM (2016). Balance of the Sexes: Addressing Sex Differences in Preclinical Research. Yale J Biol Med.

[20] Rist MJ (2017). Metabolite patterns predicting sex and age in participants of the Karlsruhe Metabolomics and Nutrition (KarMeN) study. PLoS One.

[21] Trabado S (2017). The human plasma-metabolome: Reference values in 800 French healthy volunteers; impact of cholesterol, gender and age. PLoS One.

[22] Soeters MR (2012). Characterization of D-3-hydroxybutyrylcarnitine (ketocarnitine): an identified ketosis-induced metabolite. Metabolism.

[23] Krajcovicova-Kudlackova M, Simoncic R, Bederova A, Babinska K, Beder I (2000). Correlation of carnitine levels to methionine and lysine intake. Physiol Res.

[24] Newgard CB (2009). A branched-chain amino acid-related metabolic signature that differentiates obese and lean humans and contributes to insulin resistance. Cell Metab.

[25] Drake KJ, Sidorov VY, McGuinness OP, Wasserman DH, Wikswo JP (2012). Amino acids as metabolic substrates during cardiac ischemia. Exp Biol Med (Maywood).

[26] Zieve L, Lyftogt C, Raphael D (1986). Ammonia toxicity: comparative protective effect of various arginine and ornithine derivatives, aspartate, benzoate, and carbamyl glutamate. Metab Brain Dis.

[27] Popolo A, Adesso S, Pinto A, Autore G, Marzocco S (2014). L-Arginine and its metabolites in kidney and cardiovascular disease. Amino Acids.

[28] Lotz C (2014). Characterization, design, and function of the mitochondrial proteome: from organs to organisms. J Proteome Res.

[29] Ma S (2015). Organization of the mammalian metabolome according to organ function, lineage specialization, and longevity. Cell Metab.

[30] Swann JR (2011). Systemic gut microbial modulation of bile acid metabolism in host tissue compartments. Proc Natl Acad Sci USA.

[31] Ussher JR, Lopaschuk GD (2009). Targeting malonyl CoA inhibition of mitochondrial fatty acid uptake as an approach to treat cardiac ischemia/reperfusion. Basic Res Cardiol.

[32] Huemer M (2014). Three new cases of late-onset cblC defect and review of the literature illustrating when to consider inborn errors of metabolism beyond infancy. Orphanet J Rare Dis.

[33] Brass EP, Stabler SP (1988). Carnitine metabolism in the vitamin B-12-deficient rat. Biochem J.

[34] Scolamiero E (2014). Maternal vitamin B12 deficiency detected in expanded newborn screening. Clin Biochem.

[35] Franconi F, Campesi I, Occhioni S, Tonolo G (2012). Sex-gender differences in diabetes vascular complications and treatment. Endocr Metab Immune Disord Drug Targets.

[36] Turer AT (2009). Metabolomic profiling reveals distinct patterns of myocardial substrate use in humans with coronary artery disease or left ventricular dysfunction during surgical ischemia/reperfusion. Circulation.

[37] Tucci S, Flogel U, Spiekerkoetter U (2015). Sexual dimorphism of lipid metabolism in very long-chain acyl-CoA dehydrogenase deficient (VLCAD-/-) mice in response to medium-chain triglycerides (MCT). Biochim Biophys Acta.

[38] Frye CA, Walf AA (2002). Changes in progesterone metabolites in the hippocampus can modulate open field and forced swim test behavior of proestrous rats. Horm Behav.

[39] D’Aquila PS (2010). Dopamine is involved in the antidepressant-like effect of allopregnanolone in the forced swimming test in female rats. Behav Pharmacol.

[40] Johnson DT, Harris RA, Blair PV, Balaban RS (2007). Functional consequences of mitochondrial proteome heterogeneity. Am J Physiol Cell Physiol.

[41] Carter AL, Stratman FW (1982). Sex steroid regulation of urinary excretion of carnitine in rats. J Steroid Biochem.

[42] Chiu KM, Schmidt MJ, Shug AL, Binkley N, Gravenstein S (1997). Effect of dehydroepiandrosterone sulfate on carnitine acetyl transferase activity and L-carnitine levels in oophorectomized rats. Biochim Biophys Acta.

[43] Ruoppolo M (2015). Female and male human babies have distinct blood metabolomic patterns. Mol Biosyst.

[44] Caterino M (2016). The proteome of methylmalonic acidemia (MMA): the elucidation of altered pathways in patient livers. Mol Biosyst.

[45] Spaziani S (2014). Insulin-like growth factor 1 receptor signaling induced by supraphysiological doses of IGF-1 in human peripheral blood lymphocytes. Proteomics.

[46] Imperlini E (2016). Mass spectrometry-based metabolomic and proteomic strategies in organic acidemias. Biomed Res Int.

[47] Scolamiero E (2015). Targeted metabolomics in the expanded newborn screening for inborn errors of metabolism. Mol Biosyst.

[48] Ruoppolo M (2014). Serum metabolomic profiles suggest influence of sex and oral contraceptive use. Am J Transl Res.

[49] Everitt, B. S., Landau, S., Leese, M. & Stahl, D. Cluster Analysis (John Wiley & Sons, Inc., 2011).

[50] Allen J (2003). High-throughput classification of yeast mutants for functional genomics using metabolic footprinting. Nat Biotechnol.

[51] Asafu-Adjei JK, Sampson AR, Sweet RA, Lewis DA (2013). Adjusting for matching and covariates in linear discriminant analysis. Biostatistics.

