# Supplementary material for: Targeted metabolomic profiling in rat tissues reveals sex differences
Source: Sci Rep. 2018 Mar 16;8:4663. doi: 10.1038/s41598-018-22869-7 (PMC5856765; doi:10.1038/s41598-018-22869-7)

Targeted metabolomic profiling in rat tissues reveals sex differences

Margherita Ruoppolo[1,2,3] Marianna Caterino[1,2,3], Lucia Albano [1,2], Rita Pecce[1], Maria Grazia De Girolamo[1,2], Daniela Crisci[1,2], Michele Costanzo[1], Luigi Milella[4], Flavia Franconi[5,6], Ilaria Campesi[5*]

Table S1 Analytical information about QC

Analyte	Low	Medium	High	Very high
**C0**	16.0 (13.8-18.3)	32.0 (28.5-35.5)	49.6 (41.1-58.2)	68.1 (60.4-75.8)
**C2**	10.1 (8.9-11.3)	14.7 (12.6-16.8)	21.0 (16.8-25.2)	27.5 (23.0-31.9)
**C3**	1.2 (1.0-1.3)	5.0 (4.5-5.6)	9.2 (7.9-10.6)	13.4 (12.0-14.9)
**C3DC**	0.0 (0.0-0.0)	0.4 (0.3-0.4)	1.1 (1.0-1.3)	2.3 (1.9-2.7)
**C4**	0.1 (0.1-0.1)	0.8 (0.6-0.9)	2.2 (1.9-2.6)	3.8 (3.4-4.3)
**C4OH**	0.1 (0.0-0.1)	0.4 (0.3-0.4)	0.7 (0.5-0.8)	1.6 (1.4-1.9)
**C5**	0.1 (0.1-0.1)	0.5 (0.4-0.6)	1.5 (1.2-1.7)	2.9 (2.5-3.2)
**C5DC**	0.0 (0.0-0.0)	0.5 (0.4-0.5)	1.0 (0.8-1.1)	2.3 (1.9-2.7)
**C5OH**	0.4 (0.4-0.5)	1.2 (1.1-1.4)	2.1 (1.8-2.5)	3.0 (2.7-3.4)
**C6**	0.0 (0.0-0.0)	0.4 (0.3-0.5)	0.8 (0.7-1.0)	2.1 (1.8-2.5)
**C8**	0.0 (0.0-0.0)	0.5 (0.4-0.6)	1.0 (0.8-1.2)	2.5 (2.1-2.9)
**C10**	0.0 (0.0-0.0)	0.5 (0.4-0.6)	1.0 (0.8-1.2)	2.6 (2.1-3.1)
**C12**	0.0 (0.0-0.0)	1.0 (0.7-1.2)	2.0 (1.5-2.5)	3.1 (2.5-3.7)
**C14**	0.0 (0.0-0.1)	0.5 (0.4-0.6)	1.4 (1.1-1.8)	2.9 (2.4-3.3)
**C16**	0.6 (0.5-0.8)	3.8 (3.3-4.4)	7.4 (6.0-8.8)	10.9 (9.4-12.4)
**C16OH**	0.0 (0.0-0.0)	0.2 (0.1-0.2)	0.7 (0.5-0.8)	1.0 (0.8-1.2)
**C18**	0.5 (0.4-0.6)	1.3 (1.1-1.4)	2.9 (2.3-3.5)	4.6 (3.9-5.3)
**C18OH**	0.0 (0.0-0.0)	0.1 (0.1-0.2)	0.6 (0.5-0.7)	0.9 (0.7-1.0)
**Ala**	458.6(416.5-500.8)	623.2 (542.7-703.7)	802.3 (701.5-903.2)	1012.1 (843.6-1180.7)
**Arg**	11.0 (7.6-14.4)	94.1 (79.7-108.5)	177.0 (139.1-214.9)	265.3 (211.6-319.1)
**Cit**	28.4 (24.2-32.6)	50.8 (44.8-56.8)	117.3 (97.4-137.39	257.1 (209.4-304.8)
**Leu**	145.3 (128.7-161.8)	232.1 (202.2- 261.9)	360.4 (320.1-400.7)	592.2 (493.0-691.3)
**Met**	23.4 (21.3-25.6)	67.8 (59.2-76.3)	154.7 (134.8-174.6)	252.7 (213.0-292.4)
**Phe**	80.5 (71.0-90.0)	178.0 (155.4-200.5)	268.6 (233.3-303.8)	379.4 (319.1-439.7)
**SUAC**	0.9 (0.7-1.1)	1.9 (1.6-2.3)	3.8 (3.2-4.4)	7.2 (5.6-8.8)
**Tyr**	58.2 (51.6-64.8)	233.5 (208.1-258.8)	403.6 (348.6-458.6)	598.8 (506.4-691.3)
**Val**	168.3 (141.5-195.0)	321.1 (284.6-357.6)	439.3 (394.8-483.8)	573.5 (482.1-665.0)

Data are expressed as µmol/L and reported as the means (confidence limits). SUAC : Succinylacetone

Table S2 Confusion matrix of the LDA class prediction

	Male liver	Female liver	Male heart	Female heart	Male kidney	Female kidney	Total
**Male liver**	**25**	0	0	0	0	0	25
**Female liver**	0	**23**	0	0	0	0	23
**Male heart**	0	0	**13**	2	0	0	15
**Female heart**	0	0	0	**15**	0	0	15
**Male kidney**	0	0	0	1	**13**	0	14
**Female kidney**	0	0	0	0	0	**14**	14
**Total**	25	23	13	18	13	14	106

In rows the given groups, while in columns the predicted groups; the numbers indicate the frequency of prediction. The numbers in bold face on the diagonal highlight the number of samples correctly predicted in each class.

Supplementary Figure S1 PCA stratified according to sex and organ. Panels A and B represent PCA score plots in male and female liver samples; Panels C and D represent PCA score plots in male and female heart samples; Panels E and F represent PCA score plots in male and female kidney samples.

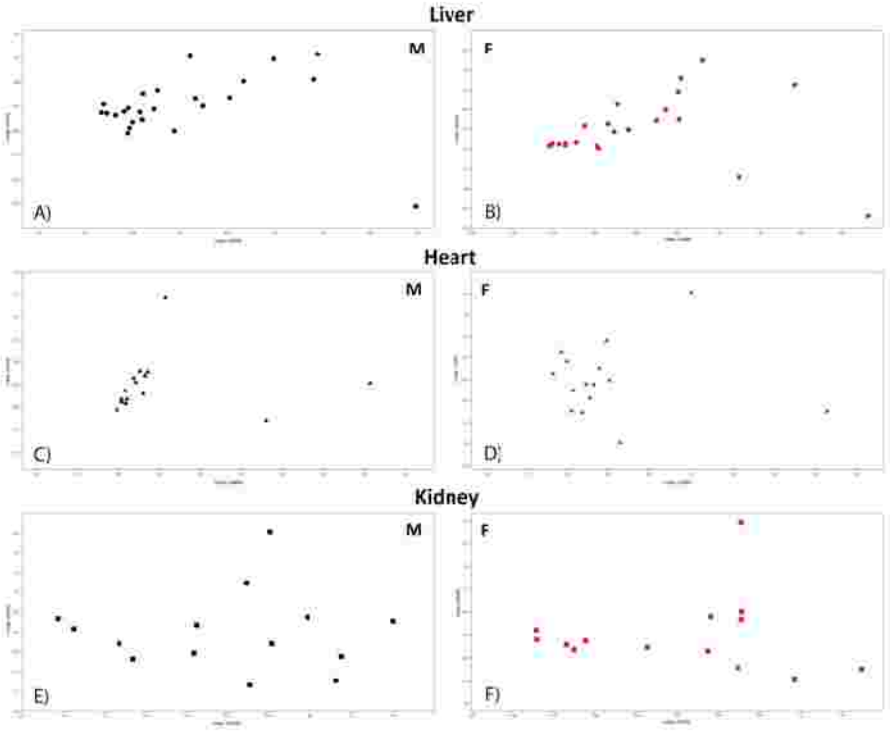

Supplement: Supplementary file 1 — Supplementary tables and figure [file 41598_2018_22869_MOESM1_ESM.pdf]
